# Metabolic and immune heterogeneity in stroke: Insights into subtype identification and biomarker discovery

**DOI:** 10.1371/journal.pone.0323172

**Published:** 2025-05-21

**Authors:** Wei Wang, Qiuyang Qian, Ziye Guo, Fangyi Lin, Weiming Lin, Zhigang Yuan, Mei Shen

**Affiliations:** 1 Rehabilitation Medicine Department, Shenzhen Longhua District People’s Hospital, Shenzhen, China; 2 Department of Critical Care Medicine, Shenzhen Longhua District People’s Hospital, Shenzhen, China; Southern Medical University, CHINA

## Abstract

Stroke is a leading cause of mortality and long-term disability worldwide, characterized by substantial molecular and clinical heterogeneity. This study aimed to investigate the metabolic and immune-related mechanisms underlying stroke subtypes using transcriptomic data and advanced computational tools. Gene expression data from two GEO datasets (GSE16561 and GSE58294) were preprocessed, batch-corrected, and integrated. Consensus clustering based on 2,752 metabolism-related genes identified three distinct subtypes with significant differences in metabolic activity. GSVA analysis revealed subtype-specific variations in key pathways, such as the citrate cycle, glycolysis, and glycosaminoglycan biosynthesis, highlighting their metabolic diversity.

## Introduction

Stroke is one of the leading causes of mortality and long-term disability worldwide, posing a significant public health challenge [1–2]. Characterized by the sudden interruption of cerebral blood flow, stroke leads to a cascade of complex pathophysiological processes, including metabolic dysregulation, inflammation, and immune responses. These multifaceted mechanisms not only contribute to the acute phase of neuronal damage but also influence long-term recovery and the potential for recurrent events. Despite substantial progress in understanding the molecular and cellular underpinnings of stroke, its heterogeneity remains a critical barrier to effective prevention and treatment strategies [3].

Recent advancements in high-throughput technologies, such as transcriptomics and metabolomics, have revolutionized our ability to dissect the molecular landscape of stroke. By integrating these data with computational approaches, researchers can identify disease subtypes with distinct molecular signatures, thereby enabling precision medicine approaches [4]. For instance, metabolism-related genes play a pivotal role in the pathophysiology of stroke, mediating critical processes such as energy production, oxidative stress, and cellular repair. However, a comprehensive exploration of how metabolic dysregulation correlates with stroke subtypes and outcomes remains limited [5].

In parallel, the immune microenvironment has emerged as a key determinant in stroke progression and recovery [6–7]. Immune cells infiltrate the brain shortly after the onset of ischemia, triggering both protective and detrimental effects [8]. The composition and activity of immune cells vary significantly among individuals, reflecting the diverse immunological landscape of stroke. Understanding this heterogeneity is essential for the development of immunomodulatory therapies [9].

Another promising approach lies in the application of machine learning techniques to uncover robust biomarkers for disease stratification and prognosis [10–11]. Algorithms such as LASSO regression and Random Forest analysis allow for the selection of key genes that may serve as predictive indicators or therapeutic targets. By integrating insights from gene expression profiles, immune cell infiltration, and metabolic pathways, it becomes possible to construct comprehensive models that delineate stroke subtypes and their associated molecular mechanisms.

This study aims to address these gaps by leveraging publicly available datasets to investigate the molecular and immunological heterogeneity of stroke. Specifically, we focus on the identification of stroke subtypes based on metabolism-related genes, the analysis of immune infiltration patterns, and the selection of hub genes using advanced computational tools. By integrating these multidisciplinary approaches, we seek to provide novel insights into the pathophysiological mechanisms underlying stroke and identify potential biomarkers for personalized medicine. Ultimately, our findings could inform the development of targeted therapeutic strategies, improving clinical outcomes for stroke patients [12].

## Methods

### 1. Data collection and processing

Gene expression data for stroke patients were obtained from the Gene Expression Omnibus (GEO) database (https://www.ncbi.nlm.nih.gov/geo/). These data were obtained using GEO database, so the approvalletter by ethics committee was not required. Two datasets, GSE16561 and GSE58294, were selected for analysis [13]. These datasets underwent data filtering, background correction, log2 transformation, and normalization. For missing values, samples with more than 10% missing data were excluded, and remaining missing values were imputed using the k-nearest neighbors (KNN) algorithm. Outliers were identified based on principal component analysis (PCA) and standardized residuals, and removed prior to downstream analysis. The datasets were then merged, and Batch correction was applied using the ComBat function from the “sva” R package. Before correction, principal component analysis (PCA) was used to assess batch effects between datasets. After ComBat adjustment, the effectiveness of batch correction was verified by visualizing PCA plots to ensure samples from different batches were well integrated [14].

### 2. Disease subtype identification

Consensus clustering was performed using a published list of 2,752 metabolism-related genes encoding known human metabolic enzymes and transporters. The goal was to classify stroke samples into distinct subtypes through consensus clustering.

The maximum number of clusters was set at 5, and a cluster consensus score threshold of > 0.8 was applied. The optimal number of clusters (k = 3) was determined based on the cumulative distribution function (CDF) plots, delta area plot, and the relative change in area under the CDF curve, ensuring stability and biological interpretability of clustering [15]. Differentially expressed genes (DEGs) between subtypes were identified using the “limma” package. Genes with an adjusted p-value < 0.05 and absolute log2 fold change (|log2FC|) > 1 were considered statistically significant.

### 3. Genomic variation analysis

Gene Set Variation Analysis (GSVA) was used as an unsupervised and non-parametric method to estimate scores for pathways or features based on transcriptomic data. Using the GSVA R package, scores corresponding to 84 metabolic features previously reported in the literature were calculated for each sample. These pathways were derived from the KEGG (Kyoto Encyclopedia of Genes and Genomes) database and represent core metabolic processes including carbohydrate metabolism, amino acid metabolism, lipid metabolism, and nucleotide metabolism [16].

### 4. Immune infiltration assessment

Various algorithms were employed to evaluate immune infiltration status. The XCELL package quantified the relative abundance of immune and stromal cells based on gene expression profiles of DFU subtypes. EPIC, ssGSEA, quanTIseq, TIMER, CIBERSORT, MCPCounter, XCELL, and ESTIMATE algorithms were used to calculate ESTIMATE scores and the relative abundance of immune cells [17].

### 5. Weighted correlation network analysis

The WGCNA package was used to construct weighted gene co-expression networks to identify gene modules associated with DFU subtypes and clinical features. First, the Pearson correlation matrix was calculated for all pairwise gene expressions. This matrix was then transformed into an adjacency matrix using a soft-thresholding power of 10. The resulting adjacency matrix was further converted into a topological overlap matrix (TOM), which measures the network connectivity of a gene with all other genes. Hierarchical clustering based on TOM dissimilarity was performed to identify gene modules using the dynamic tree cut algorithm with a minimum module size of 50.

The scale-free topology criterion was applied to determine the optimal soft threshold power. Specifically, a range of powers (1–20) was tested, and the power value of 10 was selected where the scale-free topology fitting index R² exceeded 0.85 and mean connectivity remained relatively high, ensuring a balance between network sparsity and biological relevance. A weighted adjacency matrix and topological overlap matrix were generated, followed by hierarchical clustering and tree analysis to identify modules containing more than 50 genes. Each module was visually represented with an arbitrary color, and eigengenes summarized the characteristics of each module [18].

### 6. Functional enrichment analysis

Functional enrichment analyses, including Gene Ontology (GO) and Kyoto Encyclopedia of Genes and Genomes (KEGG), were performed using the “clusterProfiler” R package to identify functions and pathways associated with central genes in the cyan module [19].

### 7. Machine learning

Stable and robust features play a crucial role in predicting the occurrence and progression of DFU. LASSO regression, a widely used linear predictive algorithm, was employed for feature selection. The optimal lambda parameter was selected via 10-fold cross-validation using the “cv.glmnet” function. For the Random Forest model, the number of trees was set to 500, and the “mtry” parameter was tuned by minimizing out-of-bag (OOB) error. These parameter settings aimed to achieve stable feature selection and robust model performance. Diagnostic performance of the selected features was visualized using Area Under the Curve (AUC) plots generated with the “pROC” R package [20].

### 8. Statistical analysis

Statistical analyses were conducted using R software (version 4.2.0). Wilcoxon tests were used for between-group comparisons, with P-values < 0.05 considered statistically significant. The major R packages and functions used include: “sva” (ComBat function for batch correction, version 3.44.0), “WGCNA” (blockwiseModules, version 1.71), “limma” (lmFit and eBayes for differential analysis, version 3.50.0), “GSVA” (gsva, version 1.42.0), “clusterProfiler” (enrichGO and enrichKEGG, version 4.2.2), “glmnet” (cv.glmnet for LASSO, version 4.1-2), “randomForest” (randomForest function, version 4.7-1.1), and “pROC” (roc function for ROC curves, version 1.18.0).

## Results

### 1. Identification of three subtypes through consensus clustering

**Fig 1:**
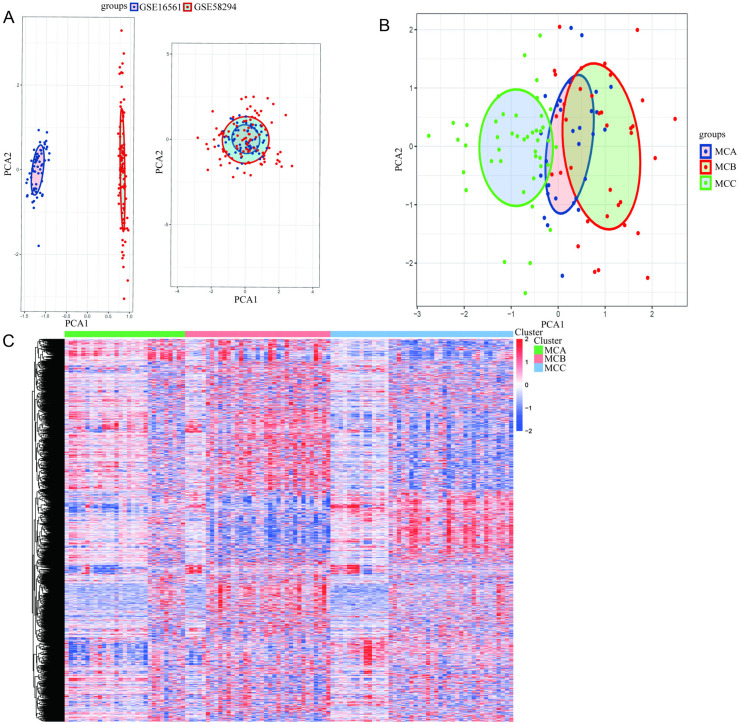
Dimensional reduction (1A), PCA clustering (1B), and heatmap of metabolic gene expression (1C) across subtypes.

Based on the reported 2,752 metabolism-related genes, datasets were merged after removing batch effects. The PCA results for the merged datasets are shown in Figs 1A and 1B. Fig 1A presents a scatter plot of the initial dataset distribution in dimensional reduction coordinates, while Fig 1B shows the clustering results of MCA, MCB, and MCC subtypes after batch correction using PCA. The subtypes were clearly separated in spatial coordinates. Fig 1C presents a heatmap of differential gene expression, highlighting distinct metabolic gene expression profiles across subtypes. These visualizations reveal significant molecular heterogeneity between stroke subtypes, providing strong clues for further exploration of underlying mechanisms.

### 2. Association between dfu subtypes and metabolic features

**Fig 2:**
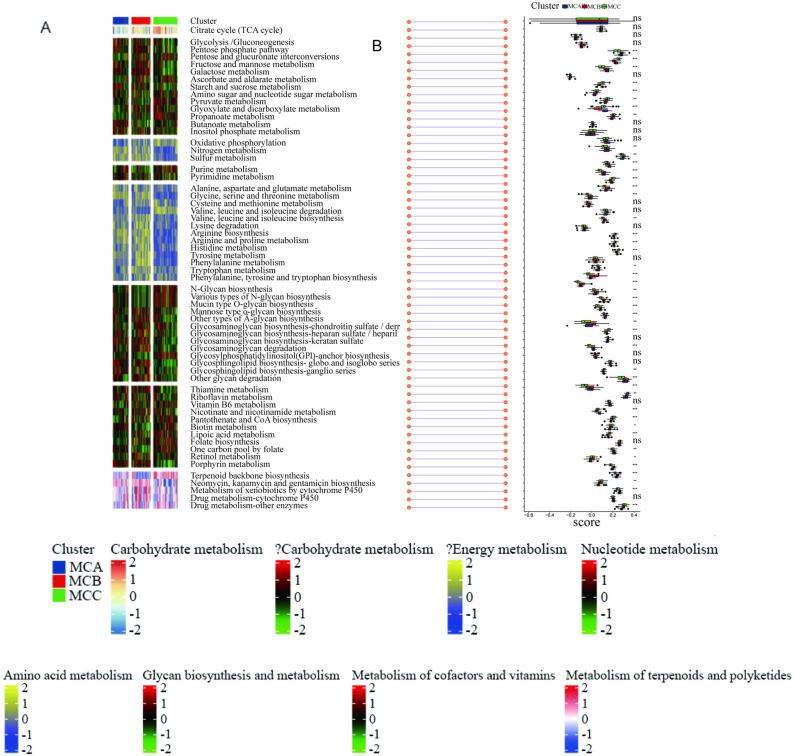
Heatmap (2A) and box plots (2B) of subtype-specific metabolic features.

Since DFU subtypes were based on metabolic genes, we explored whether different subtypes exhibited distinct metabolic characteristics. Using the “GSVA” R package, scores for 84 metabolic processes were initially measured. Differential analysis identified subtype-specific metabolic features, characterized by higher GSVA scores in relevant subtypes (Fig 2). As shown in Figure 2A, the heatmap visualization reveals distinct metabolic signatures across the MCA, MCB, and MCC subtypes. Figure 2B quantifies these differences through box plots of GSVA scores for representative pathways. 

### 3. Immune infiltration analysis across AD subtypes

**Fig 3:**
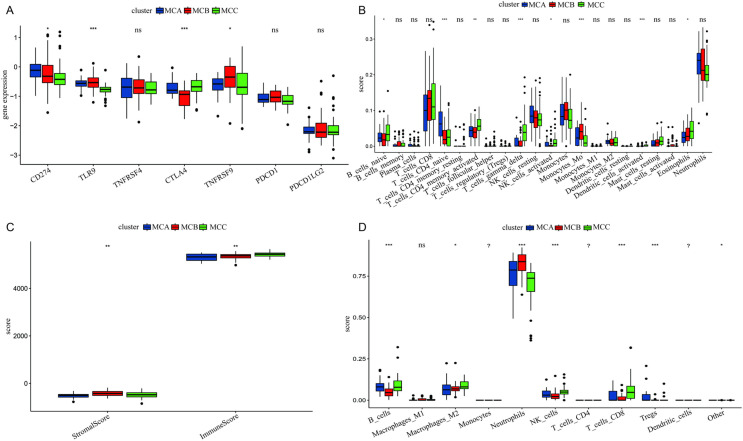
Immune checkpoint gene expression (3A), immune cell abundance (3B), stromal/immune scores (3C), and immune cell subset distribution (3D) across subtypes.

To characterize AD subtypes, immune and stromal scores were calculated using the ESTIMATE algorithm. Immune scores differed significantly among the three groups, as shown in Fig 3. Fig 3A demonstrates variability in immune checkpoint molecule expression (e.g., CD274, TNFRSF4) among MCA, MCB, and MCC subtypes, indicating active immune regulation in certain subtypes. Fig 3B highlights the abundance and activation states of various immune cells, such as B cells, T cell subsets, NK cells, and dendritic cells. Fig 3C shows significant differences in stromal and immune scores across subtypes, with some subtypes exhibiting higher immune scores, suggesting a richer immune composition in the tumor microenvironment. Fig 3D further details the distribution of specific immune cell subsets, such as M1/M2 macrophages, monocytes, neutrophils, and T cell subgroups, among subtypes. Collectively, these findings indicate significant immune microenvironment heterogeneity across subtypes, offering insights into immune regulation and potential therapeutic strategies.

### 4. Identification of Adverse DFU progression-related modules and hub genes via wGCNA

**Fig 4:**
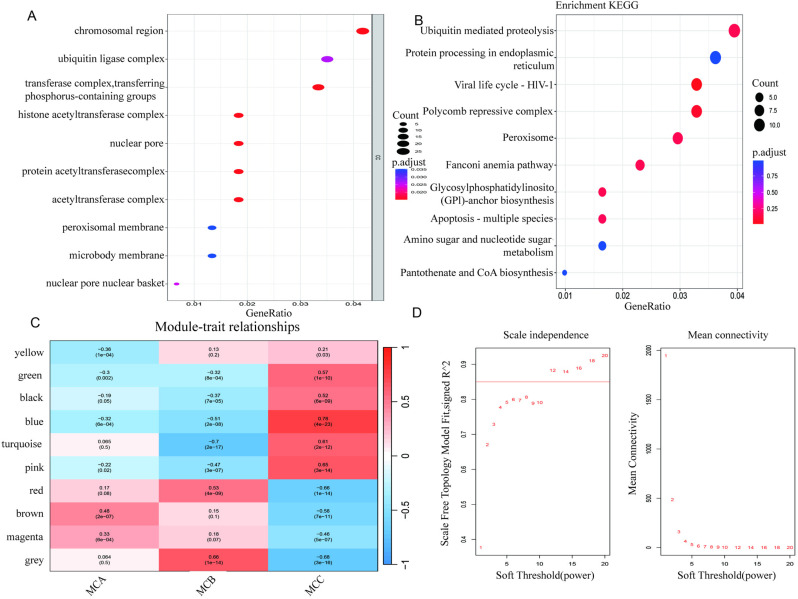
GO (4A) and KEGG (4B) enrichment, module-subtype associations (4C), and soft-threshold evaluations (4D).

WGCNA was conducted on the merged dataset to identify modules associated with adverse DFU progression. At a soft threshold power of 10, the scale-free network and connectivity achieved maximum efficiency (Fig 4D). Hierarchical clustering divided the tree into 15 gene modules, each assigned a unique color (Fig 4C). The BLUE module, containing multiple key genes, was associated with MCA and high-risk DFU indicators. Hub genes were extracted from this module using cor > 0.6 and P < 0.05. GO and KEGG enrichment analyses of hub genes (Figs 4A, 4B) revealed pathways related to protein modification, transcriptional regulation, energy metabolism, and cellular processes, providing insights into the molecular functions of stroke-associated modules.

### 5. Selection of DFU signature genes based on key modules

**Fig 5:**
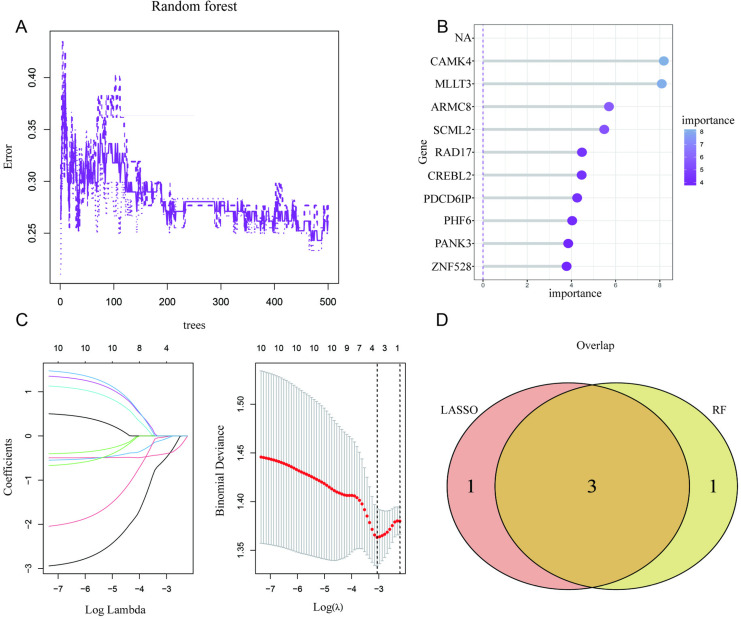
RF error curve (5A), feature importance ranking (5B), LASSO process (5C), and Venn diagram of key genes (5D).

LASSO regression was used to identify potential DFU biomarkers. Three key genes—ARMC8, CAMK4, and MLLT3—were selected based on LASSO and Random Forest (RF) algorithms. Fig 5A shows the error rate curve of the RF model with increasing decision trees. Fig 5B ranks gene importance based on RF analysis, highlighting ARMC8, CAMK4, and MLLT3. Fig 5C visualizes the LASSO regression process, identifying stable genes with the optimal Lambda. Fig 5D presents a Venn diagram of key genes overlapping between LASSO and RF methods.

### 6. Further analysis of key genes

**Fig 6:**
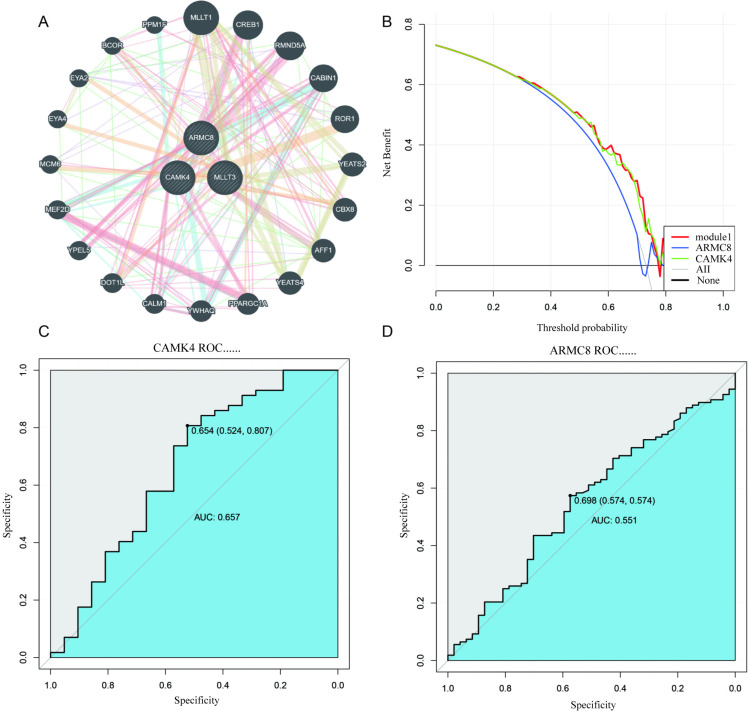
Co-expression network (6A), decision curve analysis (6B), and ROC curves for CAMK4 (6C) and ARMC8 (6D).

Fig 6A shows the co-expression network, positioning ARMC8, CAMK4, and MLLT3 as central nodes closely linked to other genes. Fig 6B illustrates decision curve analysis, where models using ARMC8 and CAMK4 achieved higher net benefits at higher threshold probabilities. Figs 6C and 6D present ROC curves for CAMK4 and ARMC8, respectively, with AUC values of 0.657 and 0.551, demonstrating moderate specificity and sensitivity for subtype distinction or outcome prediction. These findings integrate network topology, decision analysis, and diagnostic evaluation, emphasizing the clinical significance of ARMC8, CAMK4, and MLLT3.

## Discussion

This study presents several key innovations compared to prior research. First, we simultaneously integrated metabolic and immune transcriptomic signatures to identify stroke subtypes, whereas most earlier works focused on single-dimension analyses. Second, our use of consensus clustering, GSVA, WGCNA, and dual-machine learning methods (LASSO and RF) provided a multidimensional and robust strategy for subtype definition and biomarker identification. These integrative approaches represent a significant advancement toward uncovering clinically meaningful biological subtypes in stroke. Methodologically, the study is strengthened by the use of multiple independent algorithms to ensure result robustness, including consensus clustering for subtype stability, GSVA for pathway-level insight, WGCNA for gene module identification, and machine learning for biomarker selection. Batch effects were rigorously corrected, and feature selection was performed with cross-validation. These strategies not only enhance reproducibility but also provide a scalable framework applicable to other complex diseases. This study systematically investigated the molecular and immunological heterogeneity of stroke by leveraging transcriptomic data, metabolic pathway analysis, immune infiltration assessment, and machine learning approaches. The identification of three distinct stroke subtypes based on metabolism-related genes underscores the importance of metabolic pathways in stroke pathophysiology. The subtypes displayed differential activity in key metabolic pathways, including energy metabolism (e.g., the citrate cycle and glycolysis) and structural molecule synthesis (e.g., glycosaminoglycan biosynthesis). Such variations highlight the metabolic diversity among stroke patients, suggesting that targeted metabolic interventions could be developed to address specific subtype characteristics. These results provide a valuable framework for understanding the complex interplay between metabolic processes and stroke outcomes [21].

The immune microenvironment emerged as another critical determinant of stroke heterogeneity. Immune infiltration analysis revealed subtype-specific differences in the abundance and activity of immune cells, such as B cells, T cells, and macrophage subtypes (e.g., M1 and M2). Furthermore, significant variability was observed in the expression of immune checkpoint molecules, including CD274 and TNFRSF4, across subtypes. These findings suggest that different stroke subtypes may possess distinct immunoregulatory mechanisms, which could have implications for therapeutic strategies such as immune modulation or checkpoint inhibition [22]. By delineating the immunological landscape of stroke, our study provides new opportunities to personalize treatment approaches and improve patient outcomes.

The three identified stroke subtypes (MCA, MCB, and MCC) exhibited distinct metabolic and immune characteristics that align with known pathophysiological mechanisms of stroke. Specifically, the MCA subtype displayed enhanced glycolytic activity and pronounced pro-inflammatory immune signatures, likely reflecting acute ischemic damage with active immune engagement. In contrast, the MCB subtype was enriched in amino acid metabolism and showed higher proportions of anti-inflammatory cell types, suggesting a reparative or subacute stage. The MCC subtype, characterized by elevated oxidative phosphorylation, may represent a state of chronic metabolic adaptation. These findings are in line with previous reports on the metabolic and immune heterogeneity of stroke [23]. Unlike traditional clinical or imaging-based classifications, our molecularly defined subtypes provide a more integrated view of stroke biology, potentially enabling precision medicine approaches [24].

Despite the robustness of our computational pipeline, one limitation of the study lies in the relatively small sample size from public datasets, which may impact statistical power and limit the generalizability of the findings. Furthermore, the lack of prospective clinical validation restricts the immediate clinical applicability of our subtyping framework. To address this, future work should incorporate larger, multi-center cohorts and integrate transcriptomic data with clinical phenotypes and imaging features. The hub genes ARMC8, CAMK4, and MLLT3 identified in this study demonstrated diagnostic potential but also require evaluation in terms of clinical feasibility and cost-effectiveness. Detection using qPCR or immunohistochemistry is technically feasible; however, validation of sensitivity, specificity, turnaround time, and economic viability in real-world clinical settings remains essential [25]. Beyond their diagnostic value, these subtypes could also guide clinical decision-making—for instance, patients exhibiting pro-inflammatory profiles might benefit from immunomodulatory treatments, whereas those with oxidative stress phenotypes may respond better to antioxidant therapies [26]. Additionally, ARMC8, CAMK4, and MLLT3 hold promise as therapeutic targets. CAMK4 is involved in T-cell regulation and neuroinflammation, ARMC8 is linked to protein degradation and apoptosis, and MLLT3 plays a role in transcriptional regulation and neural development—all of which suggest their potential druggability pending further mechanistic validation. Compared with earlier studies that focused on single biomarkers such as IL-6 or MMP9, our integrative approach combining machine learning with co-expression analysis enabled the identification of more specific and functionally relevant gene panels. Future research should include systematic comparisons across different biomarker strategies to evaluate their predictive power and clinical utility [27].

Machine learning techniques played a pivotal role in identifying robust biomarkers for stroke subtyping and prognosis [28]. Using LASSO regression and Random Forest analysis, we identified three key genes—ARMC8, CAMK4, and MLLT3—as potential hub genes associated with adverse outcomes [29]. These genes were not only central in co-expression networks but also demonstrated diagnostic value through decision curve analysis and ROC metrics. Their involvement in metabolic and immune pathways suggests that they may serve as integrative biomarkers for stroke stratification [30]. Future studies could validate these findings in independent cohorts and explore the functional roles of these genes in stroke pathogenesis. Overall, the combination of advanced computational tools and biological insights provides a comprehensive understanding of stroke heterogeneity and offers a foundation for the development of precision medicine strategies. Despite the significant insights gained from this study, several limitations must be acknowledged [31]. First, the analysis relied on publicly available datasets, which may introduce selection bias and limit the generalizability of our findings to broader populations. Second, while we applied advanced computational methods such as GSVA, WGCNA, and machine learning, the biological mechanisms underlying the identified subtypes and key genes remain to be experimentally validated. Third, batch effects, though corrected computationally, could still contribute to residual variability, potentially influencing the robustness of subtype identification [31]. Fourth, the immune infiltration analysis was based on transcriptomic estimations rather than direct experimental measurements, which may not fully capture the complexity of the immune microenvironment. Lastly, this study focused on metabolic and immune-related pathways, potentially overlooking other important mechanisms, such as neural repair or vascular remodeling, that are equally relevant to stroke progression and recovery. Future research should include experimental validation, longitudinal data, and integrative multi-omics approaches to further elucidate the molecular landscape of stroke and refine the clinical applicability of these findings.

Despite increasing interest in the molecular underpinnings of stroke, most existing studies have examined either metabolic or immune factors in isolation, without integrating both aspects into a unified framework. Moreover, subtype classification in stroke remains largely based on clinical or imaging criteria, which often lack molecular specificity. Therefore, there is a critical need to define transcriptomic-based molecular subtypes that incorporate both metabolic and immune features to better understand patient heterogeneity and guide precision therapy. From a translational perspective, the identified molecular subtypes and hub genes offer practical implications for clinical application. Subtyping based on gene expression could complement traditional diagnostic tools to refine patient stratification. Furthermore, biomarkers such as ARMC8, CAMK4, and MLLT3 may serve as candidates for developing rapid diagnostic assays or therapeutic targets, provided they are validated in prospective clinical cohorts. These findings lay the groundwork for personalized stroke management strategies in future clinical practice. Nonetheless, one of the key limitations of this study is the absence of experimental validation. While the bioinformatics analyses have identified robust molecular subtypes and potential biomarkers, their biological relevance and clinical utility require further confirmation. In our future work, we plan to perform in vitro and ex vivo experiments to investigate the functional roles of ARMC8, CAMK4, and MLLT3 in neuronal and immune cell models under ischemic conditions. Additionally, we intend to collect clinical stroke tissue and blood samples to validate the subtype-specific expression of these genes and assess their diagnostic performance using qPCR and immunohistochemistry. These experimental efforts will bridge the gap between computational findings and clinical translation, thereby enhancing the applicability of metabolic-immune subtyping in stroke precision medicine.

## References

[1] Fu Y, Wang C, Zhang L, Ji D, Xiang A, Qi J, et al. The effectiveness of theta burst stimulation for motor recovery after stroke: a systematic review. Eur J Med Res. 2024;29(1):568. doi: 10.1186/s40001-024-02170-2 39609900 PMC11605871

[2] Isordia-Salas I, Santiago-Germán D, Jiménez-Alvarado RM, Leaños-Miranda A. Genetic Variants Associated with High Susceptibility of Premature Ischemic Stroke. J Renin Angiotensin Aldosterone Syst. 2023;2023:9002021. doi: 10.1155/2023/9002021 38025202 PMC10667057

[3] Ren J, Dong X, Nao J. Serum cystatin C is associated with carotid atherosclerosis in patients with acute ischemic stroke. Neurol Sci. 2020;41:2793–800. 32281037 10.1007/s10072-020-04383-9

[4] Meneri M, Bonato S, Gagliardi D, Comi GP, Corti S. New Insights into Cerebral Vessel Disease Landscapes at Single-Cell Resolution: Pathogenetic and Therapeutic Perspectives. Biomedicines. 2022;10(7):1693. doi: 10.3390/biomedicines10071693 35884997 PMC9313091

[5] Owolabi MO, Thrift AG, Martins S, Johnson W, Pandian J, Abd-Allah F, et al. The state of stroke services across the globe: report of World Stroke Organization–World Health Organization surveys. Int J Stroke. 2021;16(8):889–901. 33988062 10.1177/17474930211019568 PMC8800855

[6] Rahimpour S, Clary BL, Nasoohi S, Berhanu YS, Brown CM. Immunometabolism In Brain Aging and Neurodegeneration: Bridging Metabolic Pathways and Immune Responses. Aging Dis. 2024;1:1–13. 10.14336/AD.2024.1293 PMC12539545 39751865

[7] Wang Y, Liu W, Geng P, Du W, Guo C, Wang Q, et al. Role of Crosstalk between Glial Cells and Immune Cells in Blood-Brain Barrier Damage and Protection after Acute Ischemic Stroke. Aging Dis. 2023;15(6):2507–25. doi: 10.14336/AD.2023.1010 37962453 PMC11567273

[8] Endres M, Moro MA, Nolte CH, Dames C, Buckwalter MS, Meisel A. Immune Pathways in Etiology, Acute Phase, and Chronic Sequelae of Ischemic Stroke. Circ Res. 2022;130(8):1167–86. doi: 10.1161/CIRCRESAHA.121.319994 35420915

[9] Dunn CM, Kameishi S, Grainger DW, Okano T. Strategies to address mesenchymal stem/stromal cell heterogeneity in immunomodulatory profiles to improve cell-based therapies. Acta Biomater. 2021;133:114–25. doi: 10.1016/j.actbio.2021.03.069 33857693

[10] Chattopadhyay S. Decoding medical diagnosis with machine learning classifiers. Medinformatics. 2024;1:1–8.

[11] Mbarek L, Chen S, Jin A, Pan Y, Meng X, Yang X, et al. Predicting 3-month poor functional outcomes of acute ischemic stroke in young patients using machine learning. Eur J Med Res. 2024;29(1):494. doi: 10.1186/s40001-024-02056-3 39385211 PMC11466038

[12] Delgado-Chaves FM, Gómez-Vela F, Divina F, García-Torres M, Rodriguez-Baena DS. Computational Analysis of the Global Effects of Ly6E in the Immune Response to Coronavirus Infection Using Gene Networks. Genes (Basel). 2020;11(7):831. doi: 10.3390/genes11070831 32708319 PMC7397019

[13] Kadkhoda S, Darbeheshti F, Rezaei N, Azizi-Tabesh G, Zolfaghari F, Tavakolibazaz S, et al. RNA Language in Colorectal Cancer Using an Integrative Bioinformatics Approach. 2020. doi: 10.21203/rs.3.rs-19929/v1 PMC8101520 33968341

[14] Castillo-Secilla D, Gálvez JM, Carrillo-Perez F, Verona-Almeida M, Redondo-Sánchez D, Ortuno FM, et al. KnowSeq R-Bioc package: The automatic smart gene expression tool for retrieving relevant biological knowledge. Comput Biol Med. 2021;133:104387. doi: 10.1016/j.compbiomed.2021.104387 33872966

[15] Hannon BA. Independent and interacting effects of diet and genetic risk on obesity-related comorbidities. Ph.D. Dissertation, University of Illinois at Urbana-Champaign. 2020. https://www.ideals.illinois.edu/items/115723

[16] He H, Qiu Z. The importance of BUD13 in the prognosis of hepatocellular carcinoma revealed by pan-cancer analysis. Cell Mol Biol (Noisy-le-grand). 2023;69(6):41–8. doi: 10.14715/cmb/2023.69.6.7 37605593

[17] Zhao F, Wang M, Zhu J. Hypoxia-related lncRNAs to build prognostic classifier and reveal the immune characteristics of EGFR wild type and low expression of PD-L1 squamous and adenocarcinoma NSCLC. Cancer Med. 2021;10(17):6099–113. doi: 10.1002/cam4.4126 34250747 PMC8419766

[18] Hasankhani A, Bahrami A, Sheybani N, Fatehi F, Abadeh R, Ghaem Maghami Farahani H, et al. Integrated Network Analysis to Identify Key Modules and Potential Hub Genes Involved in Bovine Respiratory Disease: A Systems Biology Approach. Front Genet. 2021;12:753839. doi: 10.3389/fgene.2021.753839 34733317 PMC8559434

[19] Xu S, Hu E, Cai Y, Xie Z, Luo X, Zhan L, et al. Using clusterProfiler to characterize multiomics data. Nat Protoc. 2024;19(11):3292–320. doi: 10.1038/s41596-024-01020-z 39019974

[20] Gajowniczek K, Ząbkowski T. ImbTreeAUC: An R package for building classification trees using the area under the ROC curve (AUC) on imbalanced datasets. SoftwareX. 2021;15:100755. doi: 10.1016/j.softx.2021.100755

[21] Bale AA, Thammineni S, Bhargava R, Harley B. Hyaluronic Acid Influences Amino Acid Metabolism via Differential L‐Type Amino Acid Transporter 1 Expression in the U87‐Malignant Glioma Cell Line. Adv NanoBiomed Res. 2024;4(12): 2400107. 40017591 10.1002/anbr.202400107 PMC11864772

[22] Al-Danakh A, Safi M, Jian Y, Yang L, Zhu X, Chen Q, et al. Aging-related biomarker discovery in the era of immune checkpoint inhibitors for cancer patients. Front Immunol. 2024;15:1348189. doi: 10.3389/fimmu.2024.1348189 38590525 PMC11000233

[23] Liang J, Han R, Zhou B. Metabolic Reprogramming: Strategy for Ischemic Stroke Treatment by Ischemic Preconditioning. Biology (Basel). 2021;10(5):424. doi: 10.3390/biology10050424 34064579 PMC8151271

[24] Bonkhoff AK, Grefkes C. Precision medicine in stroke: towards personalized outcome predictions using artificial intelligence. Brain. 2022;145(2):457–75. doi: 10.1093/brain/awab439 34918041 PMC9014757

[25] Ferrari A, Neefs I, Hoeck S, Peeters M, Van Hal G. Towards Novel Non-Invasive Colorectal Cancer Screening Methods: A Comprehensive Review. Cancers (Basel). 2021;13(8):1820. doi: 10.3390/cancers13081820 33920293 PMC8070308

[26] Forman HJ, Zhang H. Targeting oxidative stress in disease: promise and limitations of antioxidant therapy. Nat Rev Drug Discov. 2021;20(9):689–709. doi: 10.1038/s41573-021-00233-1 34194012 PMC8243062

[27] Sur D, Advani S, Braithwaite D. MicroRNA panels as diagnostic biomarkers for colorectal cancer: A systematic review and meta-analysis. Front Med (Lausanne). 2022;9:915226. doi: 10.3389/fmed.2022.915226 36419785 PMC9676370

[28] Alhatemi RAJ, Savaş S. A Weighted Ensemble Approach with Multiple Pre-trained Deep Learning Models for Classification of Stroke. MEDIN. 2023;1(1):10–9. doi: 10.47852/bonviewmedin32021963

[29] Zhou W, Chen D, Li K, Yuan Z, Chen X. Multimodal photoacoustic imaging in analytic vulnerability of atherosclerosis. iRADIOLOGY. 2023;1(4):303–19. doi: 10.1002/ird3.39

[30] Saceleanu VM, Toader C, Ples H, Covache-Busuioc R-A, Costin HP, Bratu B-G, et al. Integrative Approaches in Acute Ischemic Stroke: From Symptom Recognition to Future Innovations. Biomedicines. 2023;11(10):2617. doi: 10.3390/biomedicines11102617 37892991 PMC10604797

[31] Huang J, Zhang J-L, Ang L, Li M-C, Zhao M, Wang Y, et al. Proposing a novel molecular subtyping scheme for predicting distant recurrence-free survival in breast cancer post-neoadjuvant chemotherapy with close correlation to metabolism and senescence. Front Endocrinol (Lausanne). 2023;14:1265520. doi: 10.3389/fendo.2023.1265520 37900131 PMC10602753

